# Comparative gene expression of *Wigglesworthia* inhabiting non-infected and *Trypanosoma brucei gambiense-*infected *Glossina palpalis gambiensis* flies

**DOI:** 10.3389/fmicb.2014.00620

**Published:** 2014-11-17

**Authors:** Illiassou Hamidou Soumana, Bernadette Tchicaya, Gustave Simo, Anne Geiger

**Affiliations:** ^1^UMR 177, Institut de Recherche pour le Développement-CIRADMontpellier, France; ^2^Department of Biochemistry, Faculty of Science, University of DschangDschang, Cameroon

**Keywords:** *Wigglesworthia*, tsetse fly, trypanosomes, tripartite interactions, transcriptome

## Abstract

Tsetse flies (*Glossina* sp.) that transmit trypanosomes causing human (and animal) African trypanosomiasis (HAT and AAT, respectively) harbor symbiotic microorganisms, including the obligate primary symbiont *Wigglesworthia glossinidia*. A relationship between *Wigglesworthia* and tsetse fly infection by trypanosomes has been suggested, as removal of the symbiont results in a higher susceptibility to midgut infection in adult flies. To investigate this relationship and to decipher the role of *W. glossinidia* in the fly's susceptibility to trypanosome infection, we challenged flies with trypanosomes and subsequently analyzed and compared the transcriptomes of *W. glossinidia* from susceptible and refractory tsetse flies at three time points (3, 10, and 20 days). More than 200 *W. glossinidia* genes were found to be differentially expressed between susceptible and refractory flies. The high specificity of these differentially expressed genes makes it possible to distinguish *Wigglesworthia* inhabiting these two distinct groups of flies. Furthermore, gene expression patterns were observed to evolve during the infection time course, such that very few differentially expressed genes were found in common in *Wigglesworthia* from the 3-, 10- and 20-day post-feeding fly samples. The overall results clearly demonstrate that the taking up of trypanosomes by flies, regardless of whether flies proceed with the developmental program of *Trypanosoma brucei gambiense*, strongly alters gene expression in *Wigglesworthia*. These results therefore provide a novel framework for studies that aim to decrease or even abolish tsetse fly vector competence.

## Introduction

Human African trypanosomiasis (HAT), or sleeping sickness, is a neglected vector-borne parasitic disease caused by protozoa belonging to the *Trypanosoma* genus. Two of the subspecies, *Trypanosoma brucei gambiense* and *T. b. rhodesiense*, are responsible for the chronic form of HAT in western and central Africa, and the acute form of the disease in eastern Africa, respectively (Kennedy, 2008).

Although there are drugs to treat this disease, they have limited effectiveness and produce harmful side effects (Cattand et al., 2001; Barrett, 2006). The repeated use of some drugs has led to the emergence of resistant forms of different trypanosome species (De Koning, 2001; Matovu et al., 2001). Consequently, the exploration for novel strategies must continue, including alternative vector-based strategies (Rio et al., 2004).

To be transmitted, the parasite must first establish itself within the insect midgut following an infectious blood meal, and then mature in the salivary glands or mouthparts (depending on the trypanosome species) (Vickerman et al., 1988; Van den Abbeele et al., 1999). Tsetse flies are normally refractory to trypanosome infections, and typically exhibit an infection rate of less than 50%, even under ideal laboratory conditions (Ravel et al., 2003), while field infection rates rarely exceed 10% of the fly population (Moloo et al., 1986; Frézil and Cuisance, 1994; Maudlin and Welburn, 1994). Furthermore, many infected flies fail to produce mature parasites and therefore never become infective (Moloo et al., 1986; Dukes et al., 1989; Frézil and Cuisance, 1994; Maudlin and Welburn, 1994; Jamonneau et al., 2004). This ability to acquire the parasite, facilitate its maturation, and transmit it to a mammalian host is known as vector competence.

Tsetse flies harbor 3 different symbiotic microorganisms (Aksoy, 2000), including *Sodalis glossinidius* (Cheng and Aksoy, 1999; Dale and Maudlin, 1999) and the obligate primary symbiont *Wigglesworthia glossinidia* (Aksoy, 1995a; Aksoy et al., 1995). Both are members of the Enterobacteriaceae family, inhabit the fly's gut, and are vertically transmitted to the intrauterine-developing larvae (Cheng and Aksoy, 1999). *Wigglesworthia* is localized intracellularly in specialized host cells of the anterior midgut and extracellularly in the female milk glands (Balmand et al., 2013). *Sodalis* has a broad tissular tropism and can also be found in the hemolymph, salivary glands, etc. (Cheng and Aksoy, 1999). The third symbiont, *Wolbachia* (O'Neill et al., 1993), belongs to the Rickettsiaceae family and causes cytoplasmic incompatibility in tsetse flies (Alam et al., 2011).

*S. glossinidius* has been suspected of being involved in the trypanosome establishment process (Maudlin and Ellis, 1985; Welburn et al., 1993). Epidemiological investigations conducted in several HAT foci in Cameroon have demonstrated an association between the presence of this symbiont and the establishment of trypanosome species and subspecies in the fly midgut (Farikou et al., 2010).

*Wigglesworthia* has coevolved with *Glossina* species, and develops in the fly's bacteriocytes where it encodes a plethora of vitamins and biosynthetic products that may promote host reproduction as well as fly nutrition throughout its development (Akman et al., 2002; Rio et al., 2012). In the absence of *Wigglesworthia*, vitamin supplementation of the blood meal can partially restore host fertility (Nogge, 1976, 1982). The presence of *Wigglesworthia* is necessary during the larval stages for proper immune development (Weiss et al., 2011, 2012). The presence of larval microbiota also contributes to the development of the adult peritrophic matrix (a structure that separates epithelial cells from the lumen content), which regulates immune induction following the trypanosome challenge to the fly (Weiss et al., 2013).

The role of *Wigglesworthia* in tsetse fly susceptibility to trypanosome infection remains largely unknown. A relationship between *Wigglesworthia* and trypanosome infection has been suggested, since removal of the symbiont results in higher susceptibility to midgut infection in older, non-teneral flies (Pais et al., 2008; Wang et al., 2009; Snyder and Rio, 2013). It was also previously reported that *G. morsitans morsitans* (Gmm) demonstrates much higher vector competence than *G. brevipalpis* (Gb) (Harley, 1971; Moloo and Kutuza, 1988; Moloo et al., 1994). Recently, comparison of the *Wigglesworthia* spp. genomes from Gmm and Gb revealed metabolomic differences between the primary symbiont strains harbored by these two fly species (Rio et al., 2012; Snyder and Rio, 2013). One such differences involves the chorismate, phenylalanine, and folate biosynthetic pathways, which are present in *Wigglesworthia* from Gmm but not Gb. Interestingly, African trypanosomes are auxotrophic for phenylalanine and folate; their genome, however, encodes the chorismate and folate transporters, allowing the parasite to absorb these molecules from their environment (Berriman et al., 2005; Jackson et al., 2014). This may explain why Gmm, which harbors a *Wigglesworthia* strain capable of providing the trypanosomes with the different metabolites they are unable to synthesize, is more susceptible to parasite infection than Gb (Rio et al., 2012; Snyder and Rio, 2013).

The mechanisms controlling tsetse fly infection by trypanosomes thus appear to be very complex. Nevertheless, sequencing and annotation of the *Wigglesworthia* genome from the tsetse fly (Akman et al., 2002) has enabled analysis of the gene expression profiles of these bacteria, allowing the identification of genes or pathways involved in specific traits. In this context, we have challenged a group of tsetse flies with trypanosomes and subsequently analyzed the *W. glossinidia* transcriptome from susceptible and refractory tsetse flies at three time points, in order to identify the differentially expressed genes between bacteria from these different tsetse fly groups. In addition, we were willing to perform the experiment under strictly the same conditions as those used to identify the differentially expressed genes of *Sodalis* (Hamidou Soumana et al., 2014). The investigations on *Wigglesworthia* transcriptome were, thus, performed on the samples that were previously used to analyse the *Sodalis* transcriptome.

## Materials and methods

### Ethics statement

The experimental protocols involving animals were approved by the Ethics Committee and the Veterinary Department of the Centre International de Recherche Agronomique pour le Développement (CIRAD), Montpellier, France (specific approval numbers 12TRYP03, 12TRYP04, and 12TRYP06). The experiments were conducted according to internationally recognized guidelines (French regulation).

### *T. b. gambiense* strain

The S7/2/2 *T. b. gambiense* strain used in this study was isolated in 2002 from HAT patients in the Bonon sleeping sickness focus, located in Ivory Coast (Ravel et al., 2006).

### Experimental and sampling procedures

**Figure 1:**
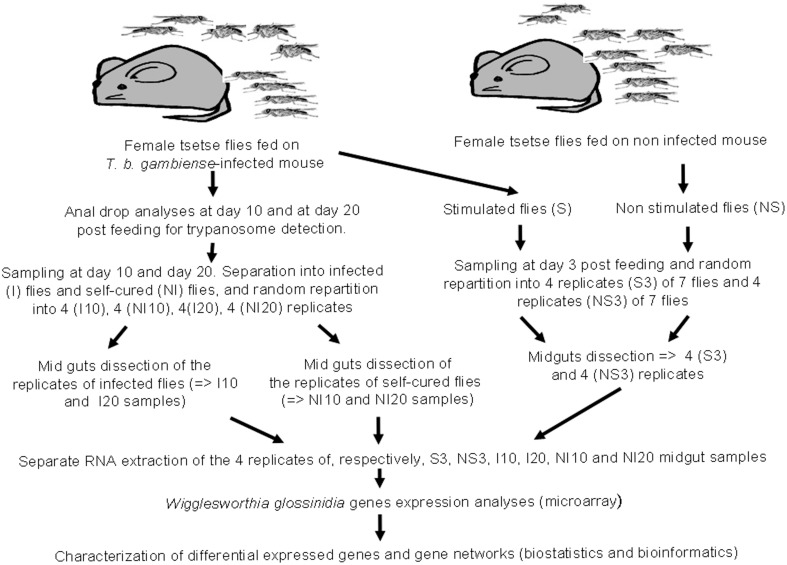
**Design depicting experimental infection, sampling procedures, and microarray data analysis**.

*Wigglesworthia* transcriptome experiments were performed on the same samples as those previously used to identify the differential expressed genes of *Sodalis* (Hamidou Soumana et al., 2014). The main experimental steps are summarized in Figure 1, and described below. All the flies under experiment were fed on infected or non-infected balb/cj mice.

A set of 100 teneral (less than 32 h old) female *G. p. gambiensis* flies from the laboratory colony were fed on non-infected mice, and a second set of 900 teneral (less than 32 h old) female flies from the same colony were fed on trypanosome-infected mice. Subsequently, non-gorged flies were removed from the experiment. After their blood meal, both gorged flies and control flies, were maintained by feeding on uninfected rabbit, 3 days per week, until the end of each experimental condition (e.g., 3, 10, and 20 days post-feeding). Sampling times were chosen according to a previously determined time course of susceptible fly infection by trypanosomes (Ravel et al., 2003), so as to compare the expression profile of *Wigglesworthia* at the different stages of the infection process. The 3-day and 10-day sampling times were selected to target differentially expressed genes involved in early events associated with trypanosome entry into the midgut and with the establishment of infection, respectively. The 20-day sampling time point was chosen to target the expression of genes involved in events occurring relatively late in trypanosome infection time course. The first set of flies (uninfected flies) was composed of four groups (four biological replicates), each of seven flies randomly chosen. Three days after their blood meal on uninfected mice, all flies from each group were dissected and the corresponding 7 midguts were pooled, yielding 4 pools of 7 midguts. These 4 biological replicates were then analyzed separately, by RNA extraction, for their transcriptome. These biological replicates are referred to as “NS 3 day samples” (NS for “non-stimulated” flies or flies not exposed to trypanosome infections).

A stabilate of *T. b. gambiense* S7/2/2 was thawed at room temperature and 0.2 ml was injected intraperitoneally into *balb/cj* mice, as described in Hamidou Soumana et al. (2014), in order to feed the second set of flies an infected blood meal. To monitor parasitemia, tail blood was examined using a phase-contrast microscope at 400× magnification. Teneral (less than 32 h old) female flies were fed on *T. b. gambiense*-infected mice displaying parasitemia levels ranging from 16 to 64 × 10^6^ parasites/ml. This set of flies was then separated into three groups. Three days after ingesting the infected blood meal, the first group was subdivided into 4 subgroups of 7 flies. All flies from each subgroup were dissected separately, and the 7 corresponding midguts were pooled, yielding 4 pools of 7 midguts (i.e., 4 biological replicates), referred to as “S 3 day samples” (S for “stimulated” flies). Following dissection, the presence of trypanosomes in the blood meal of these flies was confirmed by microscopy.

The second and third groups consist of flies that were dissected 10 and 20 days after feeding on the infected blood meal, respectively. Before dissection, the status of each fly (e.g., trypanosome infected or non-infected) was controlled by collecting anal drops from each fly, either at 10 (second group) or 20 days (third group) following their blood meal on infected mice. DNA was extracted from these anal drops using the Chelex method, as described by Ravel et al. (2003), and the presence of trypanosomes was investigated by PCR using the TBR1 and TBR2 primers (Moser et al., 1989). The identification of trypanosomes in anal drops indicates midgut infections, and flies were considered uninfected if the PCR assays were negative for the anal drops.

Based on these PCR results, tsetse flies from the second and third groups were subdivided by infection status into a subgroup of flies with trypanosome-infected midguts (e.g., a positive PCR result for the anal drop test), and a subgroup of flies without midgut infection (e.g., a negative PCR result for the anal drop test). The prevalence of infection in the second group was less than 5%, whereas it was greater than 10% in the third group. Four biological replicates were processed for each subgroup of the second and third groups, as described above for “S 3 day samples.” Samples collected 10 days after the blood meal are referred to as “I 10-day samples” and “NI 10-day samples,” and samples collected after 20 days are labeled “I 20-day samples” and “NI 20-day samples” (I and NI signify infected and non-infected flies, respectively) (Figure 1). Finally, the experiment included a total of 24 samples, consisting of 6 sets of 4 replicates each of 7 flies/7 pooled midguts; exceptionally, “I 10-day samples” and “NI 10-day samples” only grouped 3 flies, owing to the weak infection prevalence at the corresponding sampling time.

### RNA extraction

RNA was extracted from the pooled midguts of each biological replicate using TRIzol reagent (Gibco-BRL, France), according to the manufacturer's protocol. One previous study has suggested that TRIzol extraction may degrade some bacterial RNA samples (Jahn et al., 2008). The study further indicates that reproducible transcript profiling requires an investigation of RNA integrity before use, and that RNA Integrity Number values acceptable for some biological systems may not work well in other biological systems. Thus, the experiments reported here were performed in quadruplicate, and the integrity of each RNA sample (as well as the absence of contaminating DNA) was checked on an Agilent RNA 6000 Bioanalyzer. The quantification of each RNA sample was performed using the Agilent RNA 6000 Nano kit (Agilent Technologies, France).

### Oligonucleotide microarray design

A genome-wide *Wigglesworthia* transcriptome analysis from *G. p. gambiensis* was performed using the Agilent Technologies oligonucleotide microarray format (www.agilent.com). The custom-made density array (8 × 15 K format; AMADID 050087, Agilent Technologies) was designed with 60-mer oligos specific to the 673 genes of the *W. glossinidia* chromosome from *G. morsitans morsitans* (NCBI Reference Sequence: NC_016893.1) (Akman et al., 2002). For each gene, 10 different unique probes designed by Hybrigenics (Clermont-Ferrand, France) were used. The Agilent design uses the uniqueness of probe sequences as one of the criteria for probe selection, to avoid cross-hybridization with non-target genes.

The details of the array design and sample description are available at the Gene Expression Omnibus (GEO) under the accession number GPL18427. The details of the expression data are available at GEO under the accession number GSE55931.

### cDNA preparation and microarray hybridization

Cy3 dCTP direct cDNA labeling was performed with 100 ng of total RNA using the Low Input Quick Amp Labeling Kit One-Color (Agilent Technologies, France). After CyDye-labeled cDNA production, samples were carefully matched and hybridized onto the custom-made whole *W. glossinidia* Genome Oligo Microarrays. Hybridization was performed at 65°C for 17 h at 60 rpm. Microarrays were then scanned using an Agilent Technologies scanner.

### Microarray data analysis, gene ontology analysis and downstream annotation

The microarray approach has been used to analyse *W. glossinidia* gene expression, as its reliability had been validated by qPCR in our previous investigation on *Sodalis* differential gene expression (Hamidou Soumana et al., 2014). All microarray data are MIAME-compliant, and the raw and normalized data have been deposited in the MIAME-compliant GEO database (Edgar et al., 2002) (GEO accession number GSE55931). Individual microarray quality was evaluated based on the QC report, pair-wise MA plots, and box plots. The results were extracted using the Feature Extraction software 11.0.1 (Agilent Technologies). The raw data files were imported to GeneSpring GX (version 12.0, Agilent Technologies), which was used for background adjustment, quantile normalization of data (Bolstad et al., 2003; Smyth et al., 2005), log-transformation, and gene clustering for the 24 microarrays.

Gene ontology was performed only on genes with significant differential expression, using the GeneSpring database. An unpaired *t*-test was used for statistical analysis (Qin et al., 2012). A *p*-value below 0.05 was considered as statistically significant and indicates significant differences between groups (e.g., between NS 3-day samples/S 3-day samples, between I 10-day samples/NI 10-day samples, and between I 20-day samples/NI 20-day samples). The representation of each differentially expressed gene within each ontology category (including categories for molecular function, cellular component, and biological processes) was measured. A corrected *p*-value (Benjamini-Yekutelli correction) lower than 0.5 indicates over-representation of the differentially expressed genes within that particular category.

*Wigglesworthia* gene expression data were further analyzed via two-dimensional hierarchical clustering, while three-dimensional clustering of samples was analyzed by principle component analysis (PCA). Hierarchical clustering of differentially expressed genes was performed using average linkage and the Pearson-centered distance metrics (Claverie, 1999). Gene trees were created to group similar genes and to improve visualization of the data (Butte, 2002). The relationship between genes is represented by a tree in this type of clustering, where branch length reflects the degree of similarity between genes. PCA was performed within GeneSpring on infected (or stimulated) vs. non-infected (or non-stimulated) conditions at the different experimental infection times using the list of genes.

## Results

### Principal component and hierarchical clustering analysis of the *Wigglesworthia* transcription profiles

**Figure 2:**
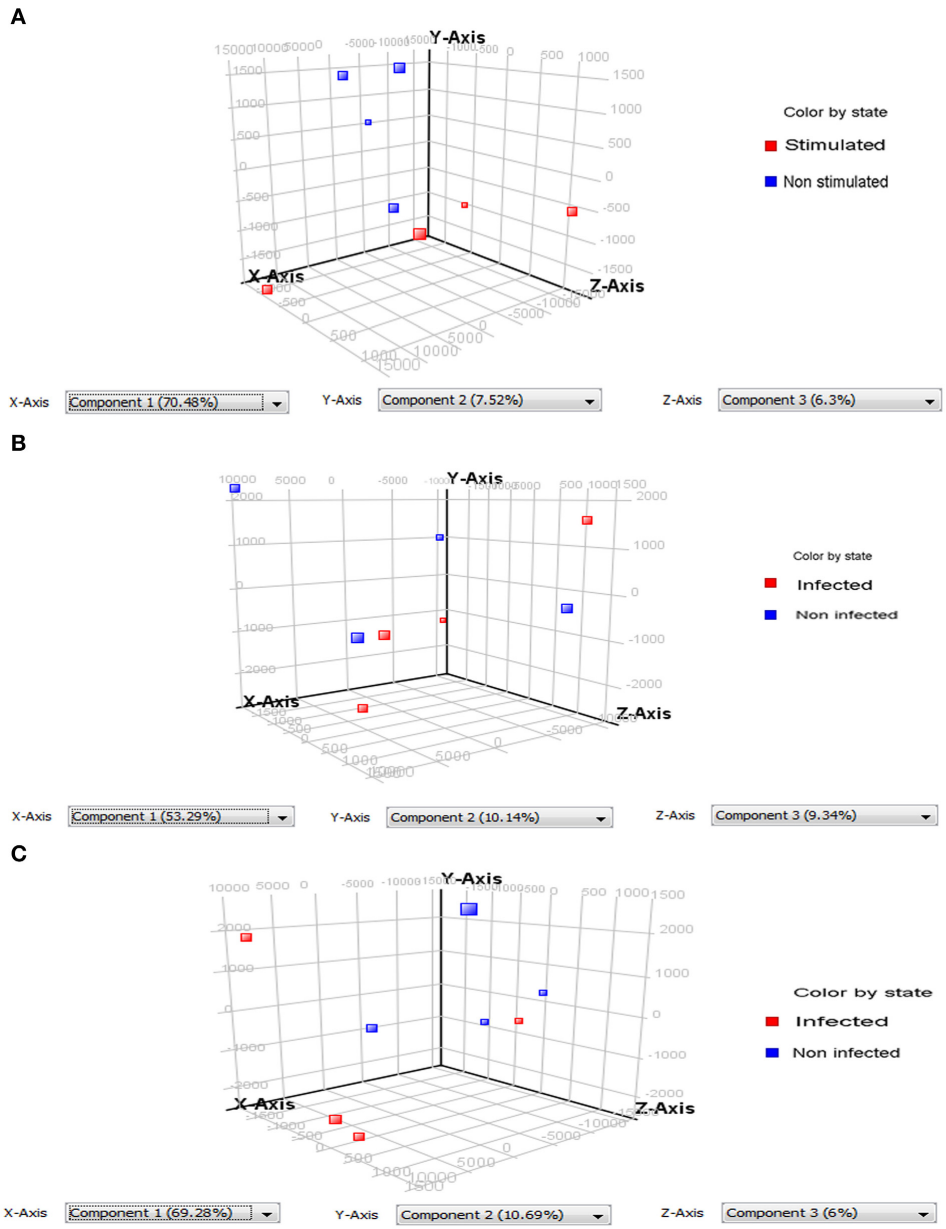
**Principle component analysis (PCA) performed on differentially expressed *Wigglesworthia* genes**. Two groups containing 4 replicates each were compared: samples from infected or stimulated flies (red squares), and from non-infected or non-stimulated flies (blue squares). **(A)** PCA on differentially expressed *Wiggleswortia* genes from stimulated and non-stimulated flies, sampled at day 3 after fly feeding. Stimulated and non-stimulated flies, were fed on trypanosome-infected and non-infected mice, respectively; **(B)** PCA on differentially expressed *Wiggleswortia* genes from infected and non-infected flies sampled at day 10 after fly feeding. **(C)** PCA on differentially expressed *Wiggleswortia* genes from infected and non-infected flies sampled at day 20 after fly feeding. For **(B,C)**, both groups of flies were fed on infected mice; the infected group comprised flies in which anal drops were trypanosome-positive, whereas the non-infected group contained flies in which anal drops were trypanosome-negative (even though they had ingested an infected blood meal).

PCA of the differentially expressed *Wigglesworthia* genes evidenced two types of results: (a) the ability to discriminate infected (or stimulated) from non-infected (or non-stimulated) flies; and (b) the existence of variability between the replicates (Figure 2). The quality of the discrimination between infected and non-infected flies however depends on the extent of the diversity between the repeats. Our results show that PCA can correctly discriminate stimulated from non-stimulated flies (3-day sampling; Figure 2A) and infected from non-infected flies at the 20-day sampling (Figure 2C), even though in each case one repeat was slightly different from the other three. The repeats were even more dispersed at the 10-day sampling, such that the infected samples could not be perfectly discriminated from the non-infected samples (Figure 2B).

**Figure 3:**
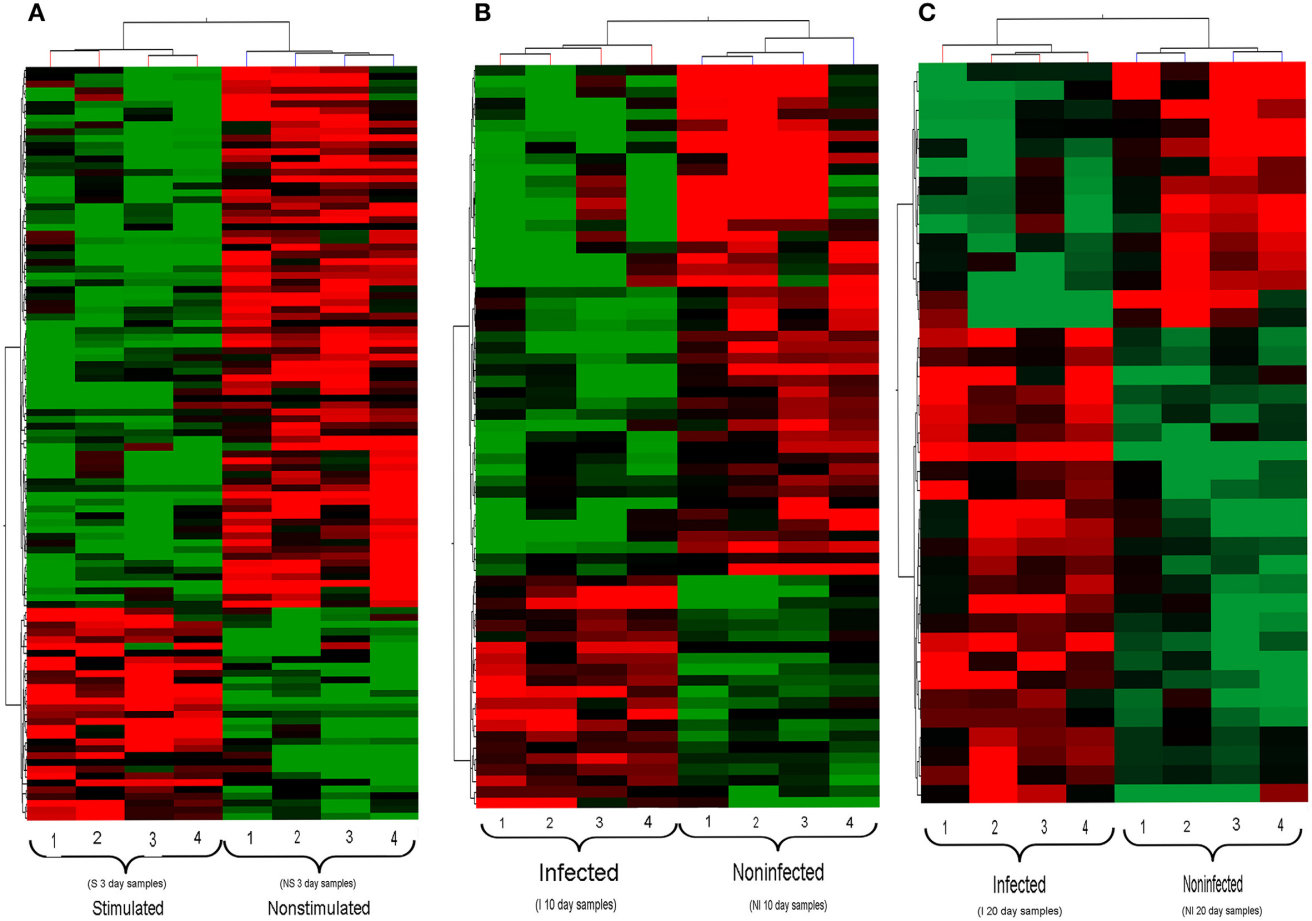
**Hierarchical clustering of *Wigglesworthia* genes**. For **(A–C)**, each group of flies is comprised of 4 replicates. **(A)** Genes from stimulated and non-stimulated tsetse flies, 3 days after ingestion of a trypanosome-infected blood meal or non-infected meal, respectively. **(B)** Genes from infected and non-infected tsetse flies, 10 days after ingestion of a trypanosome-infected blood meal. **(C)** Genes from infected and non-infected tsetse flies, 20 days after ingestion of a trypanosome-infected blood meal. The set of *Wigglesworthia* genes was extracted from the full data set (673 genes and 5115 probes) using a *t*-test (*p* < 0.05). The two-dimensional hierarchical cluster was performed on the basis of the gene expression profiles of each sample, using the GeneSpring GX software (version 12.0). Each row represents one *Wigglesworthia* gene and each column represents one *Wigglesworthia* sample. The intensity of the red and green colors, respectively up- and down-regulated genes, indicates the corresponding expression levels.

Hierarchical clustering analysis of the transcription profiles displayed by *Wigglesworthia* from stimulated and non-stimulated flies at 3 days (Figure 3A), and from infected and non-infected flies at 10 and 20 days (Figures 3B,C, respectively), clearly made *Wigglesworthia* expression profiles unique for each group. Clustering of the *Wigglesworthia* samples was based on the expression levels of: 103 genes with significant differential expression in stimulated vs. non-stimulated tsetse flies; 61 genes with significant differential expression in infected vs. non-infected tsetse flies, 10 days after ingesting the trypanosome-infected blood meal; and 38 genes with significant differential expression in infected vs. non-infected tsetse flies, 20 days after ingesting the trypanosome-infected blood meal.

### Gene expression profiling of *Wigglesworthia* in tsetse flies that are refractory and susceptible to trypanosomes

Microarray analysis of the whole *Wigglesworthia* genome expression comparing stimulated (3 days) flies to non-stimulated flies identified a total of 103 genes with significant differential expression (*p* < 0.05 according to *t*-tests, *n* = 4 Table 1). Of these genes, 73 were down-regulated and 30 were up-regulated in stimulated flies and non-stimulated flies, respectively. 79 genes exhibited a fold-change greater than 1.1 and 2 genes exhibited a fold-change greater than 1.5. 61 genes with significant differential expression (*p* < 0.05 according to *t*-tests, *n* = 4; Table 2), were identified by comparing gene expression profiles of infected and non-infected flies, 10 days after the blood meal on infected mice. 42 genes were down-regulated and 19 genes were up-regulated in infected flies and non-infected flies, respectively. 54 genes exhibited a fold-change greater than 1.1 and 6 genes exhibited a fold-change greater than 1.5. Thirty-eight *Wigglesworthia* genes were significantly differentially expressed (*p* < 0.05 according to *t*-tests, *n* = 4; Table 3) between infected and non-infected flies, 20 days after the blood meal on infected mice. 14 genes were down-regulated and 24 genes were up-regulated in infected flies and non-infected flies, respectively. 38 genes exhibited a fold-change higher than 1.1, 8 genes exhibited a fold-change greater than 1.5, 3 genes exhibited a fold-change greater than 2.0 and 1 gene (thiamine biosynthesis ThiGH complex subunit) exhibited a fold-change higher than 3.0.

**Table 1:** ***Wigglesworthia* genes displaying significant differential expression in trypanosome stimulated vs. non-stimulated flies, 3 days post-blood meal uptake**.

**Gene symbols**	**Expression**	**GenBank**	**Name**
cspE	Down	WIGMOR_0606__cspE	DNA-binding transcriptional repressor
secY	Down	WIGMOR_0186__secY	Preprotein translocase membrane subunit
groL	Down	WIGMOR_0521__groL	Cpn60 chaperonin GroEL, large subunit of GroESL
	Down	WIGMOR_0357	MarR family transcriptional regulator
sucC	Down	WIGMOR_0345__sucC	Succinyl-CoA synthetase, beta subunit
aspS	Down	WIGMOR_0121__aspS	Aspartyl-tRNA synthetase
ycfH	Down	WIGMOR_0102__ycfH	Putative metallodependent hydrolase
rpoC	Down	WIGMOR_0228__rpoC	RNA polymerase, beta prime subunit
motA	Down	WIGMOR_0035__motA	Proton conductor component of flagella motor
ybgF	Down	WIGMOR_0352__ybgF	SecB-dependent secretory protein
flhC	Down	WIGMOR_0034__flhC	Family flagellar transcriptional activator
accC	Down	WIGMOR_0138__accC	Acetyl-CoA carboxylase, biotin carboxylase subunit
yccA	Down	WIGMOR_0220__yccA	Inner membrane protein
lpxA	Down	WIGMOR_0385__lpxA	UDP-N-acetylglucosamine acyltransferase
grpE	Down	WIGMOR_0116__grpE	Pseudo
rpoZ	Down	WIGMOR_0359__rpoZ	RNA polymerase subunit omega
alaS	Down	WIGMOR_0548__alaS	Alanyl-tRNA synthetase
fusA	Down	WIGMOR_0032__fusA	GTP-binding protein chain elongation factor EF-G
	Down	WIGMOR_0040	Family flagellar basal-body P-ring formation protein
mltC	Down	WIGMOR_0079__mltC	Membrane-bound lytic murein transglycosylase C
thiL	Down	WIGMOR_0293__thiL	Thiamin-monophosphate kinase
znuA	Down	WIGMOR_0125__znuA	Periplasmic component of a high-affinity zinc uptake System
mrcB	Down	WIGMOR_0148__mrcB	Penicillin-binding protein 1B (PBP1B)
gntY	Down	WIGMOR_0476__gntY	Putative gluconate transport associate protein
tmk	Down	WIGMOR_0100__tmk	Thymidylate kinase
secA	Down	WIGMOR_0585__secA	Preprotein translocase subunit, ATPase
gyrA	Down	WIGMOR_0315__gyrA	DNA gyrase subunit A
hemF	Down	WIGMOR_0163__hemF	Coproporphyrinogen III oxidase
rplP	Down	WIGMOR_0199__rplP	50S ribosomal protein L16
rpsG	Down	WIGMOR_0031__rpsG	30S ribosomal protein S7
ftsA	Down	WIGMOR_0582__ftsA	Cell division protein
groS	Down	WIGMOR_0520__groS	Cpn10 chaperonin GroES, small subunit of GroESL
def	Down	WIGMOR_0262__def	Peptide deformylase
	Down	WIGMOR_0349	TolA family protein
putA	Down	WIGMOR_0327__putA	Transcriptional repressor/proline dehydrogenase/delta-1- pyrroline-5-carboxylate dehydrogenase
serS	Down	WIGMOR_0269__serS	Seryl-tRNA synthetase
ispG	Down	WIGMOR_0176__ispG	1-hydroxy-2-methyl-2-(E)-butenyl 4-diphosphate Synthase
hslU	Down	WIGMOR_0502__hslU	Molecular chaperone and ATPase component of HslUV protease
thiE	Down	WIGMOR_0249__thiE	Thiamin phosphate synthase
	Down	WIGMOR_0310	ncRNA
rpsR	Down	WIGMOR_0517__rpsR	30S ribosomal protein S18
sucA	Down	WIGMOR_0343__sucA	Thiamin-requiring 2-oxoglutarate decarboxylase
fliN	Down	WIGMOR_0056__fliN	Flagellar motor switching and energizing component
kdsA	Down	WIGMOR_0424__kdsA	3-deoxy-D-manno-octulosonate 8-phosphate synthase
bioC	Down	WIGMOR_0304__bioC	Malonyl-CoA methyltransferase
fliF	Down	WIGMOR_0064__fliF	Flagellar basal-body MS-ring and collar protein
	Down	WIGMOR_0083	Pyrroline-5-carboxylate reductase
holE	Down	WIGMOR_0570__holE	DNA polymerase III subunit theta
pyrI	Down	WIGMOR_0154__pyrI	Aspartate carbamoyltransferase, regulatory subunit
tyrS	Down	WIGMOR_0429__tyrS	Tyrosyl-tRNA synthetase
dapF	Down	WIGMOR_0508__dapF	Diaminopimelate epimerase
	Down	WIGMOR_0443	YhbN family protein
dapA	Down	WIGMOR_0437__dapA	Dihydrodipicolinate synthase
ydjM	Down	WIGMOR_0156__ydjM	Putative inner membrane protein
fmt	Down	WIGMOR_0263__fmt	10-formyltetrahydrofolate:L-methionyl- tRNA(fMet) N-formyltransferase
gpsA	Down	WIGMOR_0210__gpsA	Glycerol-3-phosphate dehydrogenase
recB	Down	WIGMOR_0512__recB	Exonuclease V subunit beta
pepA	Down	WIGMOR_0300__pepA	Aminopeptidase A
folD	Down	WIGMOR_0539__folD	Bifunctional 5,10-methylene-tetrahydrofolate dehydrogenase/5,10-methylene-tetrahydrofolate cyclohydrolase
rpsK	Down	WIGMOR_0183__rpsK	30S ribosomal protein S11
ileS	Down	WIGMOR_0483__ileS	Isoleucyl-tRNA synthetase
motB	Down	WIGMOR_0036__motB	Family proton-channel complex protein
ycfH	Down	WIGMOR_0102__ycfH	Putative metallodependent hydrolase
	Down	WIGMOR_0595	GltJ family glutamate/aspartate transport system permease protein
rpsI	Down	WIGMOR_0645__rpsI	30S ribosomal protein S9
rpoH	Down	WIGMOR_0076__rpoH	RNA polymerase, sigma 32 (sigma H) factor
	Down	WIGMOR_0325	tRNA
ribB	Down	WIGMOR_0545__ribB	3,4-dihydroxy-2-butanone-4-phosphate synthase
acpP	Down	WIGMOR_0097__acpP	Acyl carrier protein (ACP)
nadA	Down	WIGMOR_0624__nadA	Quinolinate synthase subunit A
pal	Up	WIGMOR_0351__pal	Peptidoglycan-associated outer membrane lipoprotein
	Up	WIGMOR_0666	tRNA
yebA	Up	WIGMOR_0126__yebA	Putative peptidase
rplX	Up	WIGMOR_0195__rplX	50S ribosomal protein L24
yggV	Up	WIGMOR_0082__yggV	dITP/XTP pyrophosphatase
guaB	Up	WIGMOR_0672__guaB	IMP dehydrogenase
	Up	WIGMOR_0558	tRNA
rplB	Up	WIGMOR_0203__rplB	50S ribosomal protein L2
hflC	Up	WIGMOR_0597__hflC	Modulator for HflB protease specific for phage lambda cII repressor
rnhA	Up	WIGMOR_0072__rnhA	Ribonuclease HI
	Up	WIGMOR_0662	tRNA
sucD	Up	WIGMOR_0346__sucD	Succinyl-CoA synthetase subunit alpha
purD	Up	WIGMOR_0245__purD	Phosphoribosylglycinamide synthetase phosphoribosylamine-glycine ligase
metG	Up	WIGMOR_0214__metG	Methionyl-tRNA synthetase
rpsC	Up	WIGMOR_0200__rpsC	30S ribosomal protein S3
flgE	Up	WIGMOR_0044__flgE	Flagellar hook protein
zapA	Up	WIGMOR_0328__zapA	Protein that localizes to the cytokinetic ring
fumC	Up	WIGMOR_0426__fumC	Fumarate hydratase
	Up	WIGMOR_0128	tRNA
	Up	WIGMOR_0135	tRNA
metK	Up	WIGMOR_0454__metK	Methionine adenosyltransferase 1
infC	Up	WIGMOR_0086__infC	Protein chain initiation factor IF-3
sucB	Up	WIGMOR_0344__sucB	Dihydrolipoyltranssuccinase
ftsH	Up	WIGMOR_0553__ftsH	Subunit of integral membrane ATP-dependent zinc metallopeptidase
	Up	WIGMOR_0134	16S ribosomal RNA
htpX	Up	WIGMOR_0111__htpX	Putative endopeptidase
	Up	WIGMOR_0237	tRNA
rnhA	Up	WIGMOR_0072__rnhA	Ribonuclease HI
mreB	Up	WIGMOR_0141__mreB	Family actin-like cell wall component
ksgA	Up	WIGMOR_0022__ksgA	S-adenosylmethionine-6-N′,N′-adenosyl (rRNA) dimethyltransferase

Wigglesworthia genes with significant differential expression, p < 0.05.

**Table 2:** ***Wigglesworthia* genes displaying significant differential expression in trypanosome infected vs. non-infected flies, 10 days post-infected blood meal uptake**.

**Gene symbols**	**Expression**	**GenBank**	**Name**
mreB	Down	WIGMOR_0141__mreB	Family actin-like cell wall component
lolE	Down	WIGMOR_0108__lolE	Membrane component of an ABC superfamily outer membrane-specific lipoprotein transporter
	Down	WIGMOR_0238	tRNA
	Down	WIGMOR_0236	Translation elongation factor Tu
yadG	Down	WIGMOR_0498__yadG	Putative ATP-binding component of an ABC superfamily transporter
	Down	WIGMOR_0160	tRNA
htpX	Down	WIGMOR_0111__htpX	Putative endopeptidase
accD	Down	WIGMOR_0463__accD	Acetyl-CoA carboxylase carboxyl transferase, beta subunit
kdsA	Down	WIGMOR_0424__kdsA	3-deoxy-D-manno-octulosonate 8-phosphate synthase
rplP	Down	WIGMOR_0199__rplP	50S ribosomal protein L16
clpX	Down	WIGMOR_0629__clpX	ATPase and specificity subunit of ClpX-ClpP ATP-dependent serine protease
secA	Down	WIGMOR_0585__secA	Preprotein translocase subunit, ATPase
hslV	Down	WIGMOR_0501__hslV	Peptidase component of the HslUV protease
	Down	WIGMOR_0369	tRNA
	Down	WIGMOR_0435	tRNA
rpsC	Down	WIGMOR_0200__rpsC	30S ribosomal protein S3
flgE	Down	WIGMOR_0044__flgE	Flagellar hook protein
gmk	Down	WIGMOR_0358__gmk	Guanylate kinase
nusG	Down	WIGMOR_0234__nusG	Transcription termination factor
yadG	Down	WIGMOR_0498__yadG	Putative ATP-binding component of an ABC superfamily transporter
sucB	Down	WIGMOR_0344__sucB	Dihydrolipoyltranssuccinase
pgk	Down	WIGMOR_0469__pgk	Phosphoglycerate kinase
mraW	Down	WIGMOR_0572__mraW	S-adenosyl-dependent methyltransferase
mreB	Down	WIGMOR_0141__mreB	Family actin-like cell wall component
acpP	Down	WIGMOR_0097__acpP	Acyl carrier protein (ACP)
mdlB	Down	WIGMOR_0567__mdlB	Putative ATP-binding component of multidrug ABC transporter
cysS	Down	WIGMOR_0538__cysS	Cysteinyl-tRNA synthetase
leuS	Down	WIGMOR_0615__leuS	Leucyl-tRNA synthetase
nusG	Down	WIGMOR_0234__nusG	Transcription termination factor
mreB	Down	WIGMOR_0141__mreB	Family actin-like cell wall component
atpA	Down	WIGMOR_0006__atpA	F1 sector of membrane-bound ATP synthase, alpha subunit
nadA	Down	WIGMOR_0624__nadA	Quinolinate synthase subunit A
bioA	Down	WIGMOR_0307__bioA	Adenosylmethionine-8-amino-7-oxononanoate aminotransferase
	Down	WIGMOR_0549	tRNA
	Down	WIGMOR_0558	tRNA
ftsA	Down	WIGMOR_0582__ftsA	Cell division protein
fbaA	Down	WIGMOR_0468__fbaA	Fructose-bisphosphate aldolase
atpD	Down	WIGMOR_0008__atpD	F1 sector of membrane-bound ATP synthase, beta subunit
flhC	Down	WIGMOR_0034__flhC	Family flagellar transcriptional activator
alaS	Down	WIGMOR_0548__alaS	Alanyl-tRNA synthetase
	Down	WIGMOR_0128	tRNA
proS	Down	WIGMOR_0145__proS	Prolyl-tRNA synthetase
rpsQ	Down	WIGMOR_0197__rpsQ	30S ribosomal protein S17
	Down	WIGMOR_0458	tRNA
	Down	WIGMOR_0134	16S ribosomal RNA
	Up	WIGMOR_0310	ncRNA
ribB	Up	WIGMOR_0545__ribB	3,4-dihydroxy-2-butanone-4-phosphate synthase
	Up	WIGMOR_0310	ncRNA
pnp	Up	WIGMOR_0564__pnp	Polynucleotide phosphorylase/polyadenylase
gyrB	Up	WIGMOR_0018__gyrB	DNA gyrase subunit B
rpsK	Up	WIGMOR_0183__rpsK	30S ribosomal protein S11
pepA	Up	WIGMOR_0300__pepA	Aminopeptidase A
flhD	Up	WIGMOR_0033__flhD	Family flagellar transcriptional activator
skp	Up	WIGMOR_0382__skp	Periplasmic chaperone
rpoH	Up	WIGMOR_0076__rpoH	RNA polymerase, sigma 32 (sigma H) factor
hemL	Up	WIGMOR_0077__hemL	Glutamate-1-semialdehyde aminotransferase
panB	Up	WIGMOR_0311__panB	3-methyl-2-oxobutanoate hydroxymethyltransferase
groS	Up	WIGMOR_0520__groS	Cpn10 chaperonin GroES, small subunit of GroESL
groL	Up	WIGMOR_0521__groL	Cpn60 chaperonin GroEL, large subunit of GroESL
truA	Up	WIGMOR_0464__truA	tRNA pseudouridine synthase A
sufS	Up	WIGMOR_0410__sufS	PLP-dependent selenocysteine lyase
prlC	Up	WIGMOR_0367__prlC	Oligopeptidase A
rlmB	Up	WIGMOR_0605__rlmB	23S rRNA Gm2251-methyltransferase
pykA	Up	WIGMOR_0648__pykA	Pyruvate kinase II

Wigglesworthia genes with significant differential expression, p < 0.05.

**Table 3:** ***Wigglesworthia* genes displaying significant differential expression in trypanosome-infected vs. non-infected flies, 20 days post-infected blood meal uptake**.

**Gene symbols**	**Expression**	**GenBank**	**Name**
cls	Down	WIGMOR_0400__cls	Cardiolipin synthase 1
rpoC	Down	WIGMOR_0228__rpoC	RNA polymerase, beta prime subunit
aroA	Down	WIGMOR_0272__aroA	3-phosphoshikimate 1-Carboxyvinyltransferase
flgC	Down	WIGMOR_0042__flgC	Flagellar component of cell-proximal portion of basal-body rod
yoaE	Down	WIGMOR_0112__yoaE	Hypothetical protein
alaS	Down	WIGMOR_0548__alaS	Alanyl-tRNA synthetase
asnS	Down	WIGMOR_0515__asnS	Asparaginyl tRNA synthetase
purF	Down	WIGMOR_0460__purF	Amidophosphoribosyltransferase
thiE	Down	WIGMOR_0249__thiE	Thiamin phosphate synthase
yadG	Down	WIGMOR_0498__yadG	Putative ATP-binding component of an ABC superfamily transporter
fusA	Down	WIGMOR_0032__fusA	GTP-binding protein chain elongation factor EF-G
groL	Down	WIGMOR_0521__groL	Cpn60 chaperonin GroEL, large subunit of GroESL
metG	Down	WIGMOR_0214__metG	Methionyl-tRNA synthetase
gpmA	Down	WIGMOR_0568__gpmA	Phosphoglyceromutase
nadA	Up	WIGMOR_0624__nadA	Quinolinate synthase subunit A
thiH	Up	WIGMOR_0253__thiH	Thiamin biosynthesis ThiGH complex subunit
pgi	Up	WIGMOR_0144__pgi	Glucosephosphate isomerase
	Up	WIGMOR_0290	tRNA
	Up	WIGMOR_0147	tRNA
rpsB	Up	WIGMOR_0374__rpsB	30S ribosomal protein S2
pepA	Up	WIGMOR_0300__pepA	Aminopeptidase A
pheS	Up	WIGMOR_0089__pheS	Phenylalanine tRNA synthetase subunit alpha
hns	Up	WIGMOR_0399__hns	Global DNA-binding transcriptional dual regulator H-NS
sufA	Up	WIGMOR_0406__sufA	Fe-S cluster assembly protein
	Up	WIGMOR_0092	Ribonuclease E
ahpC	Up	WIGMOR_0270__ahpC	Alkyl hydroperoxide reductase C22 protein
leuS	Up	WIGMOR_0615__leuS	Leucyl-tRNA synthetase
rnt	Up	WIGMOR_0432__rnt	Ribonuclease T (RNase T)
sufA	Up	WIGMOR_0406__sufA	Fe-S cluster assembly protein
pyrG	Up	WIGMOR_0413__pyrG	CTP synthetase
yidC	Up	WIGMOR_0013__yidC	Membrane insertion protein
rpoH	Up	WIGMOR_0076__rpoH	RNA polymerase, sigma 32 (sigma H) Factor
ruvC	Up	WIGMOR_0122__ruvC	Endonuclease component of RuvABC resolvasome
nrdF	Up	WIGMOR_0676__nrdF	Ferritin-like ribonucleoside-diphosphate reductase 2, beta subunit
bamA	Up	WIGMOR_0381__bamA	Outer membrane beta-barrel protein assembly factor
sufC	Up	WIGMOR_0408__sufC	Fe-S cluster assembly transport protein
skp	Up	WIGMOR_0382__skp	Periplasmic chaperone
gshA	Up	WIGMOR_0534__gshA	Gamma-glutamate-cysteine ligase
ftsY	Up	WIGMOR_0075__ftsY	Signal recognition particle protein

Wigglesworthia genes with significantl differential expression, p < 0.05.

**Figure 4:**
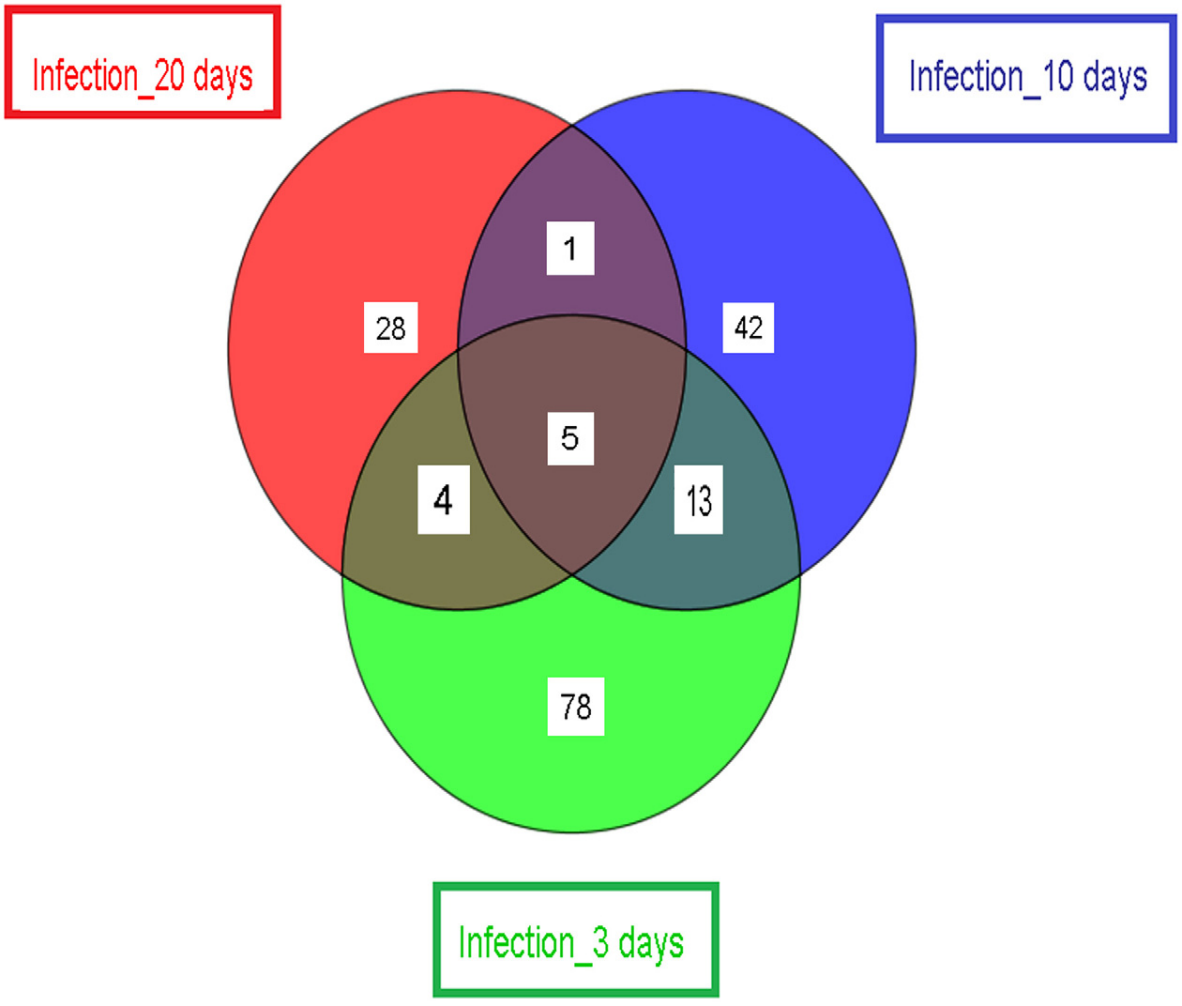
**Schematic representation showing the number of significant differentially expressed *Wigglesworthia* genes**. Samples are from day 3 (stimulated/non-stimulated flies) and days 10 and 20 (infected/non-infected flies). The number of differentially expressed genes (specific to the sampling time point) is displayed, as well as the number of differentially expressed genes shared by two or more samples.

Finally, we compared the significantly differentially expressed *Wigglesworthia* genes identified by *t*-test in the infected (or stimulated) vs. non-infected (or non-stimulated) tsetse flies at the three selected time points (3, 10, and 20 days). The results, presented in Figure 4, demonstrate that only 5 genes (WIGMOR_0521__groL: Cpn60 chaperonin GroEL; WIGMOR_0076__rpoH: RNA polymerase, sigma 32 (sigma H) factor; WIGMOR_0624__nadA: quinolinate synthase subunit A; WIGMOR_0548__alaS: alanyl-tRNA synthetase and WIGMOR_0300__pepA: aminopeptidase A) were common at all three sampling times; 78, 42, and 28 genes were specific to the 3-, 10-, and 20-day samples, respectively (Supplementary Table S1); 13 genes were found in both 3- and 10-day samples; 4 genes were found in both 3- and 20-day samples; and only one gene was found in both 10- and 20-day samples (Supplementary Table S1).

### GO annotation of the list of differentially expressed genes

We hypothesized that some of the differentially expressed genes would be involved in downstream molecular pathways that are important for maintaining susceptibility or refractoriness to trypanosome infection. To identify which types of global cellular processes or specific molecular functions are responsive, the list of differentially expressed genes obtained comparing the different conditions (stimulated/non-stimulated, infected/non-infected) from the three time points (3, 10, and 20 days) was annotated using the GO database (Blake and Harris, 2008). Each of the genes was assigned to molecular function, cellular component, and biological processes categories as designated by the GO database. Those GO categories significantly over-represented in the list of differentially expressed genes match a greater number of differentially expressed genes than would be expected by chance.

A significant over-representation of genes involved in “binding and metabolic processes” was identified in the Gene Ontology annotation of differentially expressed genes from 3-day samples (Table 4). This includes genes that encode proteins involved in protein synthesis as well as chaperonins (Cpn60 and Cpn10), which were all down-regulated in stimulated flies. The categories “developmental processes,” “morphogenesis,” and “cellular processes” displayed the greatest proportion of differentially expressed genes in the 10-day samples (Table 5), among which both chaperonins (Cpn60 and Cpn10) were up-regulated in infected flies. Surprisingly, no significant enrichment was obtained from differentially expressed *Wigglesworthia* genes in the 20-day fly samples.

**Table 4:** **Enriched Gene Ontology terms of differentially expressed genes^a^ in trypanosome-stimulated vs. non-stimulated flies, 3 days post-blood meal uptake**.

**GO accession**	**GO Term^b^**	***p*-Value**	**Corrected *p*-value^c^**	***Wigglesworthia* genes**
GO:0044267	Cellular protein metabolic process	6.505E-4	0.2539	rpsK, rplP, rpsC, rplB, fmt, rpsG, fusA, rpsR, groS, groL, rpsI, infC
GO:0030554	Adenyl nucleotide binding	7.432E-4	0.2539	groS, groL, ftsH, secA
GO:0032559	Adenyl ribonucleotide binding	7.432E-4	0.2539	groS, groL, ftsH, secA
GO:0043170	Macromolecule metabolic process	2.837E-4	0.2539	rpsK, rplP, rpsC, rplB, ksgA, rpoC, fmt, rpsG, fusA, rpoZ, recB, groS, groL, ftsH, rpsI, rnhA, rpoH, infC, rpsR
GO:004328				
GO:0005524	ATP binding	7.432E-4	0.2539	groS, groL, ftsH, secA
GO:0044238	Primary metabolic process	6.480E-4	0.25	pyrI, rpsK, rplP, rpsC, rplB, ksgA, rpoC, fmt, gyrA, rpsG, fusA, rpoZ, recB, rpsR, groS, groL, ftsH, nadA, rpsI, rnhA, rpoH, infC, tmk
GO:0005515	Protein binding	3.805E-4	0.25	groL, secA
|GO:0045308				
GO:0019538	Protein metabolic process	6.859E-4	0.25	rpsK, rplP, rpsC, rplB, fmt, rpsG, fusA, rpsR, groS, groL, ftsH, rpsI, infC
|GO:0006411				
GO:0071704	Organic substance metabolic process	8.857E-4	0.2689	pyrI, rpsK, rplP, rpsC, rplB, ksgA, rpoC, fmt, gyrA, rpsG, fusA, rpoZ, recB, rpsR, groS, groL, ribB, ftsH, nadA, rpsI, rnhA, rpoH, infC, tmk
GO:0044260	Cellular macromolecule metabolic	0.0012	0.3325	rpsK, rplP, rpsC, rplB, ksgA, rpoC, fmt, rpsG, fusA, rpoZ, rpsR, groS, groL, rpsI, rpoH, infC
|GO:0034960	Process			
GO:0001883	Purine nucleoside binding	0.0030	0.4902	fusA, groS, groL, ftsH, secA

a *This set of genes was extracted from the full data set (673 genes and 5115 probes) using a t-test (p < 0.05)*.

b *GO terms that were overrepresented in the set of differentially expressed genes*.

c *P-value after application of the Benjamini-Yekutelli correction (P < 0.5)*.

**Table 5:** **Enriched Gene Ontology terms of differentially expressed genes^a^ in trypanosome-infected vs. non-infected flies, 10 days post-infected blood meal uptake**.

**GO accession**	**GO Term^b^**	***p*-Value**	**Corrected *p*-value^c^**	***Wigglesworthia* genes**
GO:0048856	Anatomical structure development	1.508E-4	0.0555	mreB
GO:0048869	Cellular developmental process	1.508E-4	0.0555	mreB
GO:0032989	Cellular component morphogenesis	1.508E-4	0.0555	mreB
GO:0009653	Anatomical structure morphogenesis	1.508E-4	0.0555	mreB
GO:0044767	Single-organism developmental process	1.508E-4	0.0555	mreB
GO:0000902	Cell morphogenesis	1.508E-4	0.0555	mreB
|GO:0007148				
|GO:0045790				
|GO:0045791				
GO:0009987	Cellular process	0.001	0.3449	atpA, atpD, mreB, gyrB, rpsK, rpsQ, rplP, rpsC, gmk, flgE, truA, pgk, hslV, groS, groL, ribB, ftsA, nadA, clpX, pykA, rpoH
|GO:0008151				
|GO:0050875				
GO:0032502	Developmental process	0.001	0.3656	mreB

a *This set of genes was extracted from the full data set (673 genes and 5115 probes) using a t-test (p < 0.05)*.

b *GO terms that were overrepresented in the set of differentially expressed genes*.

c *P-value after application of the Benjamini-Yekutelli correction (P < 0.5)*.

## Discussion

*W. glossinidia*, the obligate symbiont of the tsetse fly, is involved in a large portion of physiological events in tsetse, including fly susceptibility or refractoriness to trypanosome infection. Several mechanisms have previously been reported, such as the modification of tsetse fly immunity or the supply of different nutrients (Pais et al., 2008; Wang et al., 2009; Rio et al., 2012; Snyder and Rio, 2013).

Presently, very few is known on the involvement of *Wigglesworthia* in tsetse flies vector competence, thus the reason of our study, a global transcriptomic approach which is the first one on the *W. glossinidia* gene differential expression. The analyses were performed at 3, 10, and 20 days post-feeding, in order to target differentially expressed genes involved in early events associated with trypanosome entry into the midgut (3 days sampling), with the establishment of infection (10 days sampling), and with events occurring relatively late in trypanosome infection time course, respectively.

Among the 673 *Wigglesworthia* genes, we identified (over the three sampling points) approximately 200 genes with significant expression changes in stimulated vs. non-stimulated flies (3-day sampling) and in infected vs. refractory flies (10- and 20-day samplings). The differences in the levels of gene expression were usually relatively weak (1.1–1.7-fold over- or under-expression), they nevertheless were statistically significant, suggestive of a biologically meaningful variation.

Only five genes were found in common when the list of genes showing modified expression in *Wigglesworthia* from 3-day samples was compared to those from 10- and 20-day samples. This result suggests the occurrence of highly complex and specific interactions that evolve during the time spent following the fly's challenge with trypanosomes.

PCA of the differentially expressed *Wigglesworthia* genes appears as a rather poor powerful tool for distinguishing stimulated from non-stimulated flies, as well as infected from non-infected flies. In contrast, clustering analysis of the gene expression data from the samples resulted in a hierarchical tree that discriminates stimulated from non-stimulated flies (3 days post-infected blood meal), and infected from non-infected flies (10 and 20 days post-infected blood meal). This clearly indicates that the expression of the *Wigglesworthia* genes not only evolves with time spent after the fly's infected blood meal (e.g., 3, 10, or 20 days), but that it also depends on the fly's status (stimulated or non-stimulated; infected or self-cured). These results are in line with those of Hamidou Soumana et al. (2014), who noted that trypanosome-responsive *Sodalis glossinidius* genes interact in well-defined patterns during the infection time course, making it possible to distinguish flies that are susceptible from those that are refractory to parasite infection.

Many of the *Wigglesworthia* genes that are down-regulated in trypanosome-stimulated and infected flies are involved in protein synthesis, such as the genes encoding ribosomal proteins, polymerases, elongation factors, etc. This was a somewhat surprising finding, as some of these proteins may be involved in pathogen survival (Nandan et al., 2002). Strikingly, we also observed that the expression of Cpn60 chaperonin GroEL, Cpn10 chaperonin GroES, and ncRNA (as well as genes implicated in the transport of bacterial toxins and in the synthesis of thiamine) were down-regulated in *Wigglesworthia* from stimulated flies (3 days post-infected blood meal), whereas the expression of the same three genes was up-regulated in *Wigglesworthia* from infected flies (10 days post-infected blood meal).

Interestingly, it has been reported that acute infection of mammalian cells with several types of viruses often results in the induction of heat-shock protein expression (Santoro, 1994), as observed for the up-regulation of chaperone proteins 24 h after infection with DENV (Chen et al., 2011). These studies support our findings for infected tsetse flies (10 days), in which two up-regulated genes (Cpn60 and Cpn10) from *Wigglesworthia* encode proteins involved in protein folding. Along these lines, the expression of a 60 kDa chaperone in the fly midgut was first described by Aksoy (1995b) and was reported once again by other researchers in 2002 (Haines et al., 2002). Several hypotheses have been made by Haines et al. (2002) which could explain the overexpression of genes encoding these chaperones:

- Chaperones may be required for bacterial survival in the hostile environment of the tsetse midgut.
- In other obligate endosymbionts, non-chaperonin activities have been reported for chaperones among which prevention of disassembly of invading microbes (Filichkin et al., 1997), protection from proteolytic degradation (Evans et al., 1992). In this context, *Wigglesworthia* may secrete chaperones that could bind proteins produced by the tsetse fly, thus protecting the trypanosome from the fly immune system.

*Enterobacter aerogenes*, for example, produces a chaperone that functions as an insect toxin, which contributes to paralyzing the ant-lion's insect prey (Yoshida et al., 2001; Haines et al., 2002). This chaperone contains four key residues (Val 100, Asn 101, Asp 338, and Ala 471) that are crucial for toxicity. The *Wigglesworthia* chaperone possesses three (Val 100, Asn 101, Ala 471) out of four of these crucial residues. This observation suggests that the *W. glossinidia* chaperones (Haines et al., 2002) (which have 86% homology with the *E. aerogenes* chaperone) could also function as toxins involved in the attrition process of trypanosomes during the early steps of their developmental cycle (occuring after the first 3 days following the infected blood meal).

Down-regulation of chaperone genes 3 days post-infected blood meal could allow further development of infection. This demonstrates the intricacy of regulations by chaperones, which depends on the time spent since the infected blood meal.

The development of trypanosome infection is complex, and several different molecules could be involved. For example, we have found that Δ(1)-pyrroline-5-carboxylate (P5C) dehydrogenase was down-expressed in tsetse flies stimulated by trypanosomes (3 days after an infected blood meal). The two-step oxidation of proline is catalyzed by proline oxidase and P5C dehydrogenase, to produce P5C and glutamate. When exogenous proline is supplied by the activities of proline oxidase and P5C reductase (conversion of P5C to proline), then impairment of P5C dehydrogenase activity can causes the P5C-proline cyclization. This proline is oxidized by the proline oxidase-FAD complex that delivers electrons to the electron transport chain and to O_2_, leading to mitochondrial reactive oxygen species (ROS) over-production. Coupled activity of proline oxidase and P5C dehydrogenase is therefore important for maintaining ROS homeostasis. In *Trypanosoma cruzi*, proline is involved in a variety of biological processes that are essential for pathogenesis (Contreras et al., 1985; Homsy et al., 1989; Martins et al., 2009).

In the case of tsetse flies that were fed on an infected blood meal, down-regulation of their P5C dehydrogenase could be due to a restriction in proline production at day 3 upon trypanosome ingestion. Furthermore, this may decrease the production of proline-rich proteins like tsetse EP (Haines et al., 2010) (involved in immunity).

The TolA (Gaspar et al., 2000) and UDP-N-Acetyl-glucosamine Acyltransferase are enzymes involved in the biosynthetic pathway of the lipopolysaccharide membrane, which acts as a barrier to the entry of antibacterial compounds (Nikaido, 1989). The *Glossina* scavenger peptidoglycan receptor PGRP-LB, plays a role in the detection and elimination of midgut trypanosomes (Wang et al., 2009). Furthermore, the virulence of some bacteria was shown to depend on secreted and cell wall peptidoglycan-associated virulence factors (Zawadzka-Skomial et al., 2006). At 3 days post-infected blood meal, the TolA and UDP-N-Acetyl-glucosamine Acyltransferase genes were down-regulated in *Wigglesworthia* from trypanosome-stimulated flies. This could favor a decrease in the stimulation of the tsetse immune response and, in turn, promote further establishment of trypanosomes.

Other proteins have been shown to be down-regulated in flies fed on infected blood meal, such as glycolytic enzymes. A number of these proteins, including glyceraldehyde-3-phosphate dehydrogenases (GAPDH), have been found to exhibit non-glycolytic functions which contribute to the ability of several bacterial pathogens to invade tissues (Boyle and Lottenberg, 1997; Tunio et al., 2010). In fact, viral infection usually causes the stimulation of glycolytic enzyme production in the midguts of *Aedes aegypti* infected with chikungunya and dengue-2 viruses (Tchankouo-Nguetcheu et al., 2010). The glycolytic enzyme GAPDH is also involved in the energetic metabolism of bloodstream trypanosomes, and can be considered as a virulence factor (Cronín et al., 1989). However, all of these processes do not likely occur in trypanosome-stimulated flies, since the glycolytic enzymes were shown to be down-regulated 3 days after flies were challenged with trypanosomes.

Therefore, our results show that trypanosome ingestion strongly alters gene expression of *Wigglesworthia*, even though the flies do not proceed with the developmental program of *T. b. gambiense*.

Ten days following an infected blood meal, the expression of several genes, such as quinolinate synthase, were down-regulated in *Wigglesworthia* from infected flies (as compared to non-infected flies). This enzyme allows the synthesis of quinolinic acid, a toxic molecule that has been detected in the central nervous system of patients displaying AIDS and meningitis (Eads et al., 1997).

In previous investigations, Weiss et al. (2011) showed that *Wigglesworthia* must be present during the immature larval stages for the tsetse fly immune system to develop and function properly at the adult stage. The artificial elimination of *Wigglesworthia* from larvae will compromise the fly immune system development throughout the development of the flies and the adult tsetse flies will display an immature immune system. In turn susceptibility to gut trypanosome infection in adult flies will be increased; in contrast, adult flies carrying *Wigglesworthia* will be highly resistant (Wang et al., 2009).

In addition, it has been suggested that differences in the susceptibility between Gmm and Gb are in turn caused by differences in the shikimate biosynthetic capabilities (phenylanine, folate and chorismate) of the *Wigglesworthia* strain they harbor (Rio et al., 2012). Our analyses failed to reveal such a role of the corresponding pathway in the vector competence of *G. p. gambiensis* for *T. b. gambiense*. Indeed, genes involved in this pathway were not found to be differentially expressed in stimulated flies at 3 days or in infected flies at 10 days, whereas some genes were found to be down-expressed (such as aro A) in infected flies at 20 days post-feeding.

Our study showed that, in trypanosomes stimulated flies (3 days post-infected bloodmeal), the expression of some *Wigglesworthia* genes are downregulated. This observation means that, despite its crucial role in fly resistance to trypanosome (as demonstrated by Wang et al., 2009; Weiss et al., 2011), the resistance level can be modulated by external factors which could favor further establishment of trypanosome. In the case of infected flies one of these external factors could be the trypanosome itself, against which the symbiont was unable to protect its host fly.

So the involvement of *Wigglesworthia* symbionts and fly immune system in tsetse fly infection by trypanosomes seems to be very complex; it is necessary but not sufficient to ensure fly resistance as demonstrated by the success of fly (carrying *Wigglesworthia*) infections performed out by number of investigators.

At the 3-day sampling time, we compared the gene expression of *Wigglesworthia* harbored within flies that ingested a non-infected blood meal to that of the symbiont of flies that ingested an infected blood meal. The blood of mice infected by trypanosomes may be quite different from non-infected mouse blood, as it transports a high concentration of trypanosomes but also may contain a range of other molecules (trypanosome-secreted proteins and other metabolites, mice reactive or degradation products, etc.). Thus, the question: besides the trypanosome itself, do these molecules, if any, that are not present in the non-infected blood meal ingested by control flies, contribute to alter the gene expression of the symbiont from flies having got the infected meal? In which extend? There is no response to this question. However, and whatever it may be, the altered gene expression of the symbiont from flies fed upon an infected meal is directly caused by the trypanosome presence, and possibly via molecules that it produced (or induced) during its development within the mammalian host. This question does not raise regarding the 10- and 20-day samples, as both groups of flies under comparison ingested the same first infected blood meal. Despite this similarity, the gene expressions differed once the flies of one group became trypanosome-infected (susceptible flies) and that of the second group self-cured the ingested trypanosomes (refractory flies).

Surprisingly, even genes encoding ribosomal proteins were differentially expressed; more surprisingly was that some of these genes were up-regulated whereas others were down-regulated. For example, in stimulated flies (S3) vs. non-stimulated flies (NS3), 50S ribosomal protein L2 and L24, and 30S ribosomal protein S3 were over-expressed whereas 50S ribosomal protein L16, and 30S ribosomal protein S7, S9, S11, and S18 were down regulated. Similar results were recorded especially in infected flies vs. refractory flies in day 10 samples (I10 vs. NI10). Similar results have been reported by Wang et al. (2013) on roots of *Arabidopsis* either phosphate- or iron-deficient vs. control roots. The authors considered the resulting alteration in ribosome composition as “*a mechanism by which plants adapt to changing environmental conditions*.” In our “model,” the stress is caused by the fly ingested trypanosomes, and results, among others, in differences in expression of genes encoding ribosomal proteins. Are these modifications involved in *W. glosssinidia* adaptation to changing environment caused by trypanosome invasion?

Finally, our results demonstrate that some genes considered as house-keeping genes are differentially expressed although their expression usually showed little variation. Fly infection cannot be compared with a classical physiological progression which could possibly account for the induction of unusual variations, even in the expression of such genes. Some of these genes also encode proteins displaying alternative functions (GAPDH, for example); alternative gene expression regulation may therefore occur regarding these genes.

The final objective of the global investigation we have undertaken is to decipher the molecular cross-talk between the tsetse fly, its symbionts, *Sodalis* and *Wigglesworthia*, and the trypanosome, in order to identify genes controlling crucial steps of fly infection by the parasite. The present study is the second step of this global investigation we have begun with the differential expression of *Sodalis* genes performed on the same biological samples. The levels of *Wiggleworthia* differentially expressed genes were lower than those recorded for *Sodalis* differentially expressed genes. Possibly the “flexibility” of gene expression is lesser in *Wigglesworthia* then in other bacteria; as an obligate symbiont, its fundamental physiological characteristics cannot be drastically modified otherwise it would no more fulfill its crucial role allowing fly survival. Nevertheless, differential expression of *Wigglesworthia* genes has been recorded, the corresponding genes identified and GO annotation performed. The results clearly demonstrate fly infection by trypanosomes to affect the symbiont transcriptomic machinery. Further investigations are necessary to assess the involvement of some of these differentially expressed genes in the parasite development process, and to evaluate their significance in the frame of an anti-vector competence strategy for combatting sleeping sickness.

### Conflict of interest statement

The authors declare that the research was conducted in the absence of any commercial or financial relationships that could be construed as a potential conflict of interest.
