# Supplementary material for: Comparative gene expression of Wigglesworthia inhabiting non-infected and Trypanosoma brucei gambiense-infected Glossina palpalis gambiensis flies
Source: Front Microbiol. 2014 Nov 17;5:620. doi: 10.3389/fmicb.2014.00620 (PMC4233935; doi:10.3389/fmicb.2014.00620)
Supplement: Supplementary file 1 [file Table1.PDF]

Supplementary Table S1. The significant Wigglesworthia differentially expressed genes selected by t-test between the stimulated (or infected) and nonstimulated (or noninfected) tsetse flies at the three sampling times (3, 10, and 20 days after fly feeding) were compared

genes found to be specific to 3-day samples

TargetID	Name
WIGMOR_0606__cspE	DNA-binding transcriptional repressor
WIGMOR_0186__secY	preprotein translocase membrane subunit
WIGMOR_0357	MarR family transcriptional regulator
WIGMOR_0345__sucC	succinyl-CoA synthetase, beta subunit
WIGMOR_0121__aspS	aspartyl-tRNA synthetase
WIGMOR_0102__ycfH	putative metallodependent hydrolase
WIGMOR_0035__motA	proton conductor component of flagella motor
WIGMOR_0352__ybgF	SecB-dependent secretory protein
WIGMOR_0034__flhC	family flagellar transcriptional activator
WIGMOR_0138__accC	acetyl-CoA carboxylase, biotin carboxylase subunit
WIGMOR_0220__yccA	inner membrane protein
WIGMOR_0385__lpxA	UDP-N-acetylglucosamine acyltransferase
WIGMOR_0116__grpE	pseudo
WIGMOR_0359__rpoZ	RNA polymerase subunit omega
WIGMOR_0040	family flagellar basal-body P-ring formation protein
WIGMOR_0079__mltC	membrane-bound lytic murein transglycosylase C
WIGMOR_0293__thiL	thiamin-monophosphate kinase
WIGMOR_0125__znuA	periplasmic component of a high-affinity zinc uptake System
WIGMOR_0148__mrcB	penicillin-binding protein 1B (PBP1B)
WIGMOR_0476__gntY	putative gluconate transport associate protein
WIGMOR_0100__tmK	thymidylate kinase
WIGMOR_0315__gyrA	DNA gyrase subunit A
WIGMOR_0163__hemF	coproporphyrinogen III oxidase
WIGMOR_0031__rpsG	30S ribosomal protein S7
WIGMOR_0262__def	peptide deformylase
WIGMOR_0349	TolA family protein

WIGMOR_0327__putA	transcriptional repressor/proline dehydrogenase/delta-1-pyrroline-5-carboxylate dehydrogenase
WIGMOR_0269__serS	seryl-tRNA synthetase
WIGMOR_0176__ispG	1-hydroxy-2-methyl-2-(E)-butenyl 4-diphosphate Synthase
WIGMOR_0502__hslU	molecular chaperone and ATPase component of HslUV protease
WIGMOR_0517__rpsR	30S ribosomal protein S18
WIGMOR_0343__sucA	thiamin-requiring 2-oxoglutarate decarboxylase
WIGMOR_0056__fliN	flagellar motor switching and energizing component
WIGMOR_0424__kdsA	3-deoxy-D-manno-octulosonate 8-phosphate synthase
WIGMOR_0304__bioC	malonyl-CoA methyltransferase
WIGMOR_0064__fliF	flagellar basal-body MS-ring and collar protein
WIGMOR_0083	pyrroline-5-carboxylate reductase
WIGMOR_0570__holE	DNA polymerase III subunit theta
WIGMOR_0154__pyrI	aspartate carbamoyltransferase, regulatory subunit
WIGMOR_0429__tyrS	tyrosyl-tRNA synthetase
WIGMOR_0508__dapF	diaminopimelate epimerase
WIGMOR_0443	YhbN family protein
WIGMOR_0437__dapA	dihydrodipicolinate synthase
WIGMOR_0156__ydjM	putative inner membrane protein
WIGMOR_0263__fmt	10-formyltetrahydrofolate:L-methionyl- tRNA(fMet) N-formyltransferase
WIGMOR_0210__gpsA	glycerol-3-phosphate dehydrogenase
WIGMOR_0512__recB	exonuclease V subunit beta
WIGMOR_0539__foldD	bifunctional 5,10-methylene-tetrahydrofolate dehydrogenase/5,10-methylene-tetrahydrofolate cyclohydrol
WIGMOR_0183__rpsk	30S ribosomal protein S11
WIGMOR_0483__ileS	isoleucyl-tRNA synthetase
WIGMOR_0036__motB	family proton-channel complex protein
WIGMOR_0102__ycfH	putative metallodependent hydrolase
WIGMOR_0595	GltJ family glutamate/aspartate transport system permease protein
WIGMOR_0645__rpsI	30S ribosomal protein S9
WIGMOR_0325	tRNA
WIGMOR_0097__acpP	acyl carrier protein (ACP)
WIGMOR_0351__pal	peptidoglycan-associated outer membrane lipoprotein

WIGMOR_0666	tRNA
WIGMOR_0126__yebA	putative peptidase
WIGMOR_0195__rplX	50S ribosomal protein L24
WIGMOR_0082__yggV	dITP/XTP pyrophosphatase
WIGMOR_0672__gnaB	IMP dehydrogenase
WIGMOR_0203__rplB	50S ribosomal protein L2
WIGMOR_0597__hflC	modulator for HflB protease specific for phage lambda cII repressor
WIGMOR_0072__rnhA	ribonuclease HI
WIGMOR_0662	tRNA
WIGMOR_0346__sucD	succinyl-CoA synthetase subunit alpha
WIGMOR_0245__purD	phosphoribosylglycinamide synthetase phosphoribosylamine-glycine ligase
WIGMOR_0328__zapA	protein that localizes to the cytokinetic ring
WIGMOR_0426__fumC	fumarate hydratase
WIGMOR_0135	tRNA
WIGMOR_0454__metK	methionine adenosyltransferase 1
WIGMOR_0086__infC	protein chain initiation factor IF-3
WIGMOR_0553__ftsH	subunit of integral membrane ATP-dependent zinc metallopeptidase
WIGMOR_0134	16S ribosomal RNA
WIGMOR_0237	tRNA
WIGMOR_0022__ksgA	S-adenosylmethionine-6-N',N'-adenosyl (rRNA) dimethyltransferase

genes found to be specific to 10-day samples

TargetID	Name
WIGMOR_0108__lolE	membrane component of an ABC superfamily outer membrane-specific lipoprotein transporter
WIGMOR_0238	tRNA
WIGMOR_0236	translation elongation factor Tu
WIGMOR_0160	tRNA
WIGMOR_0463__accD	acetyl-CoA carboxylase carboxyl transferase, beta subunit
WIGMOR_0424__kdsA	3-deoxy-D-manno-octulosonate 8-phosphate synthase
WIGMOR_0629__clpX	ATPase and specificity subunit of ClpX-ClpP ATP-dependent serine protease
WIGMOR_0501__hslV	peptidase component of the HslUV protease

WIGMOR_0369	tRNA
WIGMOR_0435	tRNA
WIGMOR_0358__gmk	guanylate kinase
WIGMOR_0234__nusG	transcription termination factor
WIGMOR_0498__yadG	putative ATP-binding component of an ABC superfamily transporter
WIGMOR_0469__pgk	phosphoglycerate kinase
WIGMOR_0572__mraW	S-adenosyl-dependent methyltransferase
WIGMOR_0097__acpP	acyl carrier protein (ACP)
WIGMOR_0567__mdlB	putative ATP-binding component of multidrug ABC transporter
WIGMOR_0538__cysS	cysteinyl-tRNA synthetase
WIGMOR_0615__leuS	leucyl-tRNA synthetase
WIGMOR_0006__atpA	F1 sector of membrane-bound ATP synthase, alpha subunit
WIGMOR_0307__bioA	adenosylmethionine-8-amino-7-oxononanoate aminotransferase
WIGMOR_0549	tRNA
WIGMOR_0468__fbaA	fructose-bisphosphate aldolase
WIGMOR_0008__atpD	F1 sector of membrane-bound ATP synthase, beta subunit
WIGMOR_0034__flhC	family flagellar transcriptional activator
WIGMOR_0145__proS	prolyl-tRNA synthetase
WIGMOR_0197__rpsQ	30S ribosomal protein S17
WIGMOR_0458	tRNA
WIGMOR_0134	16S ribosomal RNA
WIGMOR_0307__bioA	adenosylmethionine-8-amino-7-oxononanoate aminotransferase
WIGMOR_0564__pnp	polynucleotide phosphorylase/polyadenylase
WIGMOR_0018__gyrB	DNA gyrase subunit B
WIGMOR_0183__rpsK	30S ribosomal protein S11
WIGMOR_0033__flhD	family flagellar transcriptional activator
WIGMOR_0382__skp	periplasmic chaperone
WIGMOR_0077__hemL	glutamate-1-semialdehyde aminotransferase
WIGMOR_0311__panB	3-methyl-2-oxobutanoate hydroxymethyltransferase
WIGMOR_0464__truA	tRNA pseudouridine synthase A
WIGMOR_0410__sufS	PLP-dependent selenocysteine lyase

WIGMOR_0367__prlC	oligopeptidase A
WIGMOR_0605__rlmB	23S rRNA Gm2251-methyltransferase
WIGMOR_0648__pykA	pyruvate kinase II

genes found to be specific to 20-day samples

TargetID	Name
WIGMOR_0400__cls	cardiolipin synthase 1
WIGMOR_0272__aroA	3-phosphoshikimate 1-Carboxyvinyltransferase
WIGMOR_0042__flgC	flagellar component of cell-proximal portion of basal-body rod
WIGMOR_0112__yoaE	hypothetical protein
WIGMOR_0515__asnS	asparaginyl tRNA synthetase
WIGMOR_0460__purF	amidophosphoribosyltransferase
WIGMOR_0568__gpmA	phosphoglyceromutase
WIGMOR_0253__thiH	thiamin biosynthesis ThiGH complex subunit
WIGMOR_0144__pgi	glucosephosphate isomerase
WIGMOR_0290	tRNA
WIGMOR_0147	tRNA
WIGMOR_0374__rpsB	30S ribosomal protein S2
WIGMOR_0089__pheS	phenylalanine tRNA synthetase subunit alpha
WIGMOR_0399__hns	global DNA-binding transcriptional dual regulator H-NS
WIGMOR_0406__sufA	Fe-S cluster assembly protein
WIGMOR_0092	ribonuclease E
WIGMOR_0270__ahpC	alkyl hydroperoxide reductase C22 protein
WIGMOR_0615__leuS	leucyl-tRNA synthetase
WIGMOR_0432__rnt	ribonuclease T (RNase T)
WIGMOR_0413__pyrG	CTP synthetase
WIGMOR_0013__yidC	membrane insertion protein
WIGMOR_0122__ruvC	endonuclease component of RuvABC resolvasome
WIGMOR_0676__nrdF	ferritin-like ribonucleoside-diphosphate reductase 2, beta subunit
WIGMOR_0381__bamA	outer membrane beta-barrel protein assembly factor
WIGMOR_0408__sufC	Fe-S cluster assembly transport protein

WIGMOR_0382__skp	periplasmic chaperone
WIGMOR_0534__gshA	gamma-glutamate-cysteine ligase
WIGMOR_0075__ftsY	signal recognition particle protein

genes common to 3-, 10- and 20-day samples

TargetID	Name
WIGMOR_0521__groL	Cpn60 chaperonin GroEL, large subunit of GroESL
WIGMOR_0076__rpoH	RNA polymerase, sigma 32 (sigma H) factor
WIGMOR_0624__nadA	quinolinate synthase subunit A
WIGMOR_0548__alaS	alanyl-tRNA synthetase
WIGMOR_0300__pepA	aminopeptidase A

genes common to 3- and 10-day samples

TargetID	Name
WIGMOR_0585__secA	preprotein translocase subunit, ATPase
WIGMOR_0199__rplP	50S ribosomal protein L16
WIGMOR_0582__ftsA	cell division protein
WIGMOR_0520__groS	Cpn10 chaperonin GroES, small subunit of GroESL
WIGMOR_0310	ncRNA
WIGMOR_0545__ribB	3,4-dihydroxy-2-butanone-4-phosphate synthase
WIGMOR_0558	tRNA
WIGMOR_0200__rpsC	30S ribosomal protein S3
WIGMOR_0044__flgE	flagellar hook protein
WIGMOR_0128	tRNA
WIGMOR_0344__sucB	dihydrolipoyltranssuccinase
WIGMOR_0111__htpX	putative endopeptidase
WIGMOR_0141__mreB	family actin-like cell wall component

genes common to 3- and 20-day samples

TargetID	Name
WIGMOR_0228__rpoC	RNA polymerase, beta prime subunit
WIGMOR_0032__fusA	GTP-binding protein chain elongation factor EF-G
WIGMOR_0249__thiE	thiamin phosphate synthase
WIGMOR_0214__metG	methionyl-tRNA synthetase

gene common to 10- and 20-day samples

TargetID	Name
WIGMOR_0498__yadG	putative ATP-binding component of an ABC superfamily transporter

Synthase

lase
